# Social defeat drives hyperexcitation of the piriform cortex to induce learning and memory impairment but not mood-related disorders in mice

**DOI:** 10.1038/s41398-022-02151-1

**Published:** 2022-09-10

**Authors:** Hanjie Wang, Fang Li, Xuefeng Zheng, Lianghui Meng, Meiying Chen, Yuqing Hui, Yifei Li, Keman Xie, Jifeng Zhang, Guoqing Guo

**Affiliations:** 1 Department of Anatomy, Neuroscience Laboratory for Cognitive and Developmental Disorders, Medical College of Jinan University, Guangzhou, China; 2 Department of Gastroenterology, The First Affiliated Hospital, Jinan University, Guangzhou, China

**Keywords:** Neuroscience, Depression

## Abstract

Clinical studies have shown that social defeat is an important cause of mood-related disorders, accompanied by learning and memory impairment in humans. The mechanism of mood-related disorders has been widely studied. However, the specific neural network involved in learning and memory impairment caused by social defeat remains unclear. In this study, behavioral test results showed that the mice induced both learning and memory impairments and mood-related disorders after exposure to chronic social defeat stress (CSDS). c-Fos immunofluorescence and fiber photometry recording confirmed that CaMKIIα expressing neurons of the piriform cortex (PC) were selectively activated by exposure to CSDS. Next, chemogenetics and optogenetics were performed to activate PC CaMKIIα expressing neurons, which showed learning and memory impairment but not mood-related disorders. Furthermore, chemogenetic inhibition of PC CaMKIIα expressing neurons significantly alleviated learning and memory impairment induced by exposure to CSDS but did not relieve mood-related disorders. Therefore, our data suggest that the overactivation of PC CaMKIIα expressing neurons mediates CSDS-induced learning and memory impairment, but not mood-related disorders, and provides a potential therapeutic target for learning and memory impairment induced by social defeat.

## Introduction

Recently, bullying has become a widespread issue in both schools and workplaces [1, 2]. Victims of bullying tend to have poor academic and work performance [3]. The negative social experiences of these victims are known to predict psychiatric disorders such as depression, learning disabilities, low performance in memory, self-abasement, high anxiety, post-traumatic stress disorder symptoms, and even suicide [4–7]. Bullies often inflict intentional, repetitive, and persistent aggressive behavior on their victims, and victims of bullying are often in an emotional state known as social defeat [8]. The chronic social defeat stress (CSDS) model closely mimics real-life human bullying in which dominant animals bully subordinate animals [9]. In this model, animals are induced to fight for territory or food until one of them yields, with the loser showing aberrant mood-related behaviors due to physical and psychological stress. The social defeat model has become widely accepted in the study of depression-like behaviors associated with social stress [10]. Recent research has shown that CSDS contributes to learning and memory disorders in mice [4, 11–13]. However, few studies have reported the neurobiological mechanisms of learning and memory impairment induced by CSDS.

Learning and memory impairments are associated with neural circuit disorders, and the cause of learning and memory disorders may be located in different regions of the brain [14]. We found that the piriform cortex (PC) was selectively activated in mice exposed to CSDS. PC is considered a primary olfactory sensory region with known roles in emotion and memory [15, 16] and extends from rostral to dorsal in the rodent brain [17]. PC is a three-layered lamination that is characteristic of paleocortical regions [18]. Layer I is the most superficial, with a relatively sparse cell population that receives input fibers from the lateral olfactory tract. Layer II contains densely packed somata of glutamate-releasing excitatory neurons with a high level of structural plasticity. Layer III, the deepest layer, also contains lower-density excitatory neurons [19, 20]. It has extensive connections with the amygdala, entorhinal cortex, and hippocampus and plays an important role in emotion, memory, and social behavior [21]. PC is also implicated in various neurological disorders, such as epilepsy, autism spectrum disorder, Parkinson’s disease, and Alzheimer’s disease [22]. However, it remains unclear whether PC is involved in the pathological process of learning and memory impairment in CSDS mice.

The present study focused on the neural mechanisms underlying learning and memory impairments after CSDS exposure. First, we confirmed that CSDS induces learning and memory dysfunction and mood-related disorders, accompanied by the strong activation of PC CaMKIIα expressing neurons. Furthermore, chemogenetic and optogenetic activation of PC CaMKIIα expressing neurons results in learning and memory dysfunction in mice, but not mood-related disorders. Lastly, inhibition of the activation of PC CaMKIIα expressing neurons rescued CSDS-induced learning and memory impairment, while mood-related disorders were not alleviated.

## Materials and methods

### Animals

All animal experiments were conducted in accordance with the National Institutes of Health Guide for the Care and Use of Laboratory Animals and approved by the Jinan University Laboratory Animal Ethics Committee (Guangzhou, China). Male C57BL/6 J mice (6 weeks old, 18–25 g) and retired breeder male CD1 mice (7–8 months old) were purchased from Beijing Vital River Laboratory Animal Technology Limited Company (Beijing, China) and allowed to acclimatize prior to the start of the experiment. All mice were kept at a constant temperature (20–25 °C) and humidity (40–60%) in a controlled facility on a 12 h light/dark cycle (light from 8:00 to 20:00) with food and water ad libitum. To minimize possible carryover effects of the different behavioral tests, the sequence of tests was arranged from the least stressful to the most stressful [23, 24].

Age-matched mice in the same condition were randomly assigned to the control group, CSDS group, ChR2 group, hM4D(Gi) group, and hM3D(Gq) group. This was an exploratory study, thus no formal power or sample-size estimation was performed [25]. The group size was based on prior experience and literature reports on chemogenetics and optogenetics studies [26, 27].

### Acute and chronic social defeat stress

The procedures for social defeat stress have been described in previous studies [9, 28]. The protocol consisted of three stages. The first stage was the screening of aggressive CD1 intruder mice. The C57BL/6 J mice were directly placed into the home cage of the CD1 mice for 3 min, and three consecutive screening sessions were performed with different C57BL/6 J mice, once daily. Only CD1 mice that met two criteria were used: the initial attack time was less than 60 s, and an attack occurred on at least 2 consecutive days. Mice for acute social defeat stress (ASD) were exposed to a CD1 for 10 min for once. For chronic social defeat stress (CSDS), C57BL/6 J mice were exposed to different CD1 intruder mice for ten consecutive days for 10 min/day. After the 10 min social defeat, the C57BL/6 J, and aggressive male CD1 mice were divided on either side of the cage by perforated organic plastic partitions for the remaining 24 h. The same method was used to control animals during the social defeat sessions, except that the CD1 mice were replaced with the C57BL/6 J strain.

### Social interaction test (SIT)

The mice were placed in the test room to acclimatize for at least 1 h before each behavioral session. Previous work has shown that CSDS would produce social avoidance; mice can be divided into susceptible and resilient mice based on this behavioral endpoint [29]. The SIT was performed 24 h after the last social defeat using the method previously described [9, 28]. The test consisted of two 2.5 min sessions. Mice were introduced into an open field arena (40 cm length × 40 cm width × 40 cm height) with a clear-plastic cage (10 cm length × 6.5 cm width × 40 cm height) on a delineated interaction zone. The interaction zone is 8 cm wide area surrounding the clear-plastic cage. The corner zone is defined as 9 × 9 cm area for both corners opposing the clear-plastic cage. In the first “No target” trial, an empty cage was placed in the interaction zone. In the second “Target” trial, the cage of the interaction zone was placed on an unfamiliar male CD1 mouse. During the trials, the time that the C57BL/6 J mice spent in the “interaction zone” and the “corner zone” of the “No target” and “target” rails was measured using a behavior analysis system (TopScanLite Version 2.00). The SI ratio was calculated as the amount of time spent in the interaction zone with the “Target” trial divided by the amount of time spent in the interaction zone with the “No target” trial. If the SI ratio was <1, the mice were considered ‘susceptible’, and if the SI ratio was >1, the mice were considered ‘resilient’.

### Chronic restraint stress (CRS)

In the CRS experiment, mice were randomly divided into control and CRS groups [30, 31]. The CRS mice were placed in a 50 ml syringe alone (with a 1 cm hole in the wall of the cylinder for air diffusion) with the end fixed with a needle plug for 6 h per day for 21 days. The control mice remained in their original cages and were left undisturbed in the same room.

### Chronic unpredictable mild stress (CUMS)

The mice were divided into control and CUMS groups. The CUMS procedure was adopted from the previous studies [32, 33]. Briefly, CUMS mice were exposed to randomized stressors twice a day over 3 weeks, including restraint stress for 8 h, cold swimming at 4° for 5 min, foot shock for 5 min (1 mA, 5 s each time, 10 s interval), tail pinch for 3 min, wet bedding for 12 h, food and water deprivation for 12 h, 45° cage tilting for 12 h, and overnight illumination. No same stressor was applied for 2 consecutive days. The control mice were not handled with those stressors.

### Elevated plus maze test (EPM)

The anxiety-like behavior of the mice was measured using the EPM test, which was conducted 50 cm above the floor and consisted of four arms, including two closed arms (35 cm length × 5 cm width × 15 cm height), two open arms (35 cm length × 5 cm width), and a central area (5 cm length × 5 cm width) that connects the four arms. Each mouse was placed in the central area toward an open arm and allowed to explore the maze for 10 min. Entry into the arms and the time spent in the open arm was measured using a behavior analyzing system (TopScanLite version 2.00). The maze was thoroughly cleaned with 75% alcohol and dried after each test to prevent residual odors.

### Open field test (OFT)

The OFT was used to measure anxiety-like behavior [34]. Briefly, the mice were placed in the center of an OFT box (40 cm length × 40 cm width × 40 cm height) and allowed to explore the arena for 10 min. The motion trials were recorded using a camera directly above the box. The time spent in the center zone and total distance were measured using a behavior analyzing system (TopScanLite Version 2.00).

### Forced swim test (FST)

The FST was used to measure depression-like behavior. Briefly, mice were placed individually in a cylindrical tank (16 cm diameter, 28 cm height) of water maintained at 22–25 °C for 6 min, in which the depth of water was ~20 cm to ensure that the mice could not touch the bottom [35]. Before each test, the water was changed to prevent the influence of pheromones left behind by the previous mice. Locomotion was recorded from the side using a camera. Mice were considered immobile when they were floating without any other movement, and the immobility time during the last 4 min was measured with a behavior analyzing system (TopScanLite Version 2.00). After the test, the mice were dried and returned to their home cages.

### Y-maze

A Y-maze was used to assess the spatial memory of the mice as described previously [36]. The apparatus of the Y-maze was composed of three arms (40 cm length × 5 cm width × 10 cm height) at a 120° angle from each other. These arms were randomly divided into start, other, and novel arms. The start arm was the arm in which the mouse started exploring and opened in both trials, the other arm was also opened in both trials, and the novel arm was blocked in the first trial and opened in the second trial. The test consisted of two trials. In the first trial, the mice were placed in the start arm and allowed to explore the start arm and the other arm for 10 min. Then, 2 h later, a second trial was conducted. After opening the novel arm, the mice were placed back at the end of the start arm and allowed 5 min to explore, with free access to all three arms. The trials were recorded using a camera and analyzed using a behavior analyzing system (TopScanLite Version 2.00). The time spent in the novel arm was an indicator of spatial memory abilities.

### Tail suspension test (TST)

The TST was used to detect depression-like behavior, and the procedure has been described previously [37]. The animals were placed in a testing room for 1 h of acclimation. The mice were suspended by their tails with tape (~1.5 cm from the tip of the tail) in a box (20 cm length × 20 cm width × 30 cm height) with the head 5 cm from the bottom of the box. From start to finish, the TST was 6 min in length, and all the tests were recorded by a video camera and analyzed using a behavior analyzing system (TopScanLite version 2.00). The immobility time in the last 4 min (3–6 min) was determined.

### Morris water maze (MWM)

The MWM mainly determines the spatial learning of mice, and the procedure has been previously described [38, 39]. The pool for MWM was 120 cm in diameter and filled with water (22 ± 1 °C) to a depth of 40 cm. The pool was divided into four quadrants, with four objects suspended on the pool wall as close visual references. During the acquisition phase, a small round platform (12 cm in diameter) was placed 1 cm underwater in a fixed spatial position in a quadrant 20 cm from the pool wall. The water was made opaque using titanium dioxide (T164497, Biohonor, China) to ensure that the mice could not see the platform. The spatial acquisition phase consisted of 15 training trials. The mice were trained to search for the hidden platform three times per day for 5 days. Each trial started from a random quadrant that did not house the platform, with the heads of the mice facing the pool wall. Each training trial had a maximum duration of 90 s. The test interval was at least 30 min. If the mice found the platform within 90 s and stayed there for 20 s, the trial ended, and they were returned to their cage until the next trial. If the mice did not reach the platform within 90 s, the experimenter guided them to the platform and allowed them to remain there for 20 s. The latency was used for subsequent analysis. One day after the acquisition phase, the platform was removed, and the 90 s probe trial was performed. A camera recorded the time spent in the target quadrant, and the absolute time traversing the hidden platform region.

### Immunostaining and imaging

The mice were anesthetized with sodium pentobarbital (120 mg/kg) and sequentially transcardially perfused with 30 ml 0.9% NaCl, followed by 30 ml 4% paraformaldehyde (PFA) (30525-89-4, Sigma, Missouri, USA) after SIT or behavior tests for 90 min. The brains were quickly removed and fixed in 4% PFA at 4 °C overnight. After dehydration of the brains with 15% and 30% sucrose solutions (57-50-1, Sigma), they were sliced at 30 μm using a rapid sectioning cryostat (LEICA CM1900, LEICA, Weztlar, Germany).

The sections for injection site verification of the virus were rinsed for 10 min in 0.1 M PBS three times. The sections for CaMKIIα and c-Fos immunofluorescence labeling were blocked in 0.1 M PBS (P4417, Sigma) containing 5% bovine serum albumin (BSA, CAS9048-46-8, GENVIEW, Florida, USA) and 0.1% Triton X-100 (CAS9002-93-1, BIOFROXX, Germany) for 1 h. The samples were then incubated for 48 h overnight at 4 °C in 0.1 M PBS containing 1% BSA with or without (as negative control samples) primary antibodies: rabbit anti-c-Fos antibody (1:500 dilution, Rabbit mAb 2250 s, Cell Signaling Technology, Boston, USA), mouse anti-CaMKIIα antibody (1:100 dilution, ab5683, abcam, Cambridge, UK). After rinsing for 5 min in 0.1 M PBS three times, the sections were incubated with the corresponding secondary antibodies: goat anti-rabbit antibody, Alexa-fluor 488 (1:1000 dilution, A21206, Invitrogen, Carlsbad, USA) or goat anti-mouse antibody, Alexa-fluor 555 (1:1000 dilution, A31570, Invitrogen) for 2 h at ambient temperature.

Finally, the sections were mounted onto gelatin-coated slides and covered with Fluoro-Gel with DAPI (catalog no. 17985-50, Electron Microscopy Sciences, Hatfeild, UK). Fluorescent images were obtained using a laser scanning confocal microscope (TCS XP8, Leica).

### Immunohistochemistry

The section preparation method is as described above for immunofluorescence. Sections were washed with 0.1 M PBS for 3 × 10 min, then incubated in 0.3% H_2_O_2_ at room temperature for 30 min; the sections were incubated at 4° with rabbit anti-c-Fos antibody (1:500 dilution, Rabbit mAb 2250 s, Cell Signaling Technology) for 48 h; After rinsing, the sections were incubated with Biotinylated Anti-Rabbit IgG (BA1000, Vector laboratories, Newark, United States) at room temperature for 2 h; Section was washed with 0.1 M PBS for 3 × 10 min, incubated in avidin–biotin-complex (PK6100, Vector laboratories, Newark, United States) for 2 h at room temperature; Sections were rinsed, followed by 0.05% DAB (SK4100, Vector laboratories, Newark, United States) in 3% H_2_O_2_ for 5 min; after 3 × 10 min washes in PBS, the sections were mounted onto gelatin-coated slides, air-dried, dehydrated using graded ethanol, vitrification by dimethylbenzene, coverslipped with resins, and images are captured using a microscope (DM6000B, Leica).

### Injection of viral vectors and implantation of optical fiber

The surgical procedure has been previously described [27, 40, 41]. The mice were deeply anesthetized with isoflurane and positioned on stereotaxic equipment (68045, RWD, Shenzhen, China). Erythromycin eye ointment was applied to prevent corneal drying. The skin above the skull was cut to expose the skull, and small craniotomy holes (1 mm diameter) were drilled above the PC (ML: ±3.85 mm; AP: −1.58 mm; DV: −5.2 mm). Injections were performed using a micropipette connected to a nanoliter injector, and its controller (LEGATO 130, RWD, Shenzhen, China), and the speed was maintained at a rate of 0.05 μl/min.

For in vivo chemogenetic activation, AAV2/9-CaMKIIα-hM3D(Gq)-GFP (3 × 10^12^ GC/ml, PT-0525, BrainVTA, Wuhan, China) or AAV2/9-CaMKIIα-GFP (3 × 10^12^ GC/ml, PT-0290, BrainVTA) was injected unilaterally into the PC at a volume of 0.3 μl.

For in vivo chemogenetic inhibition, AAV2/9-CaMKIIα-hM4D(Gi)-GFP (3 × 10^12^ GC/ml, PT-0524, BrainVTA) or AAV2/9-CaMKIIα-GFP (3 × 10^12^ GC/ml, PT-0290, BrainVTA) were injected into the PC bilaterally at a volume of 0.3 μl for each site.

For optogenetic activation, AAV-CaMKIIα-ChR2-mCherry (3 × 10^12^ GC/ml, S0166-9-H20, TaiTool, Shanghai, China) or AAV-CaMKIIα-mCherry (3 × 10^12^ GC/ml, S0242-9-H20, TaiTool) was injected unilaterally into the PC at a volume of 0.3 μl.

For in vivo calcium signal recording, AAV-CaMKIIα-GCaMP6m (3 × 10^12^ GC/ml, S0481-9-H5, TaiTool) at a volume of 0.3 μl was injected into the PC unilaterally.

Following injection, the glass pipette was left in place for an additional 10 min and then slowly extracted to prevent virus leakage in the track.

For optogenetic activation and in vivo calcium signal recording, optic fibers (200 μm in diameter, AG-FOC, Nanjing Aoguan Biotechnology Co.Ltd, Nanjing, China) with a ceramic ferrule were implanted on the PC (ML: ±3.85 mm; AP: −1.58 mm; DV: −5.2 mm) according to previous literature [40]. Three miniature screws (1 mm diameter, 3 mm length) were fixed on the skull, and dental cement was used to connect the skull, screws, and optical fibers for structural support.

Finally, the wound was sutured, and antibiotics were applied to the surgical wound. The animals were placed under a heat lamp to recover from anesthesia. Only the mice with verified injection sites were included in the analysis.

### In vivo chemogenetic stimulation

For chemogenetic manipulations, 3 weeks after virus injection, the mice were intraperitoneally injected with CNO (3.3 mg/kg) (C0832, Sigma) 45 min before the behavioral test.

### In vivo optogenetic stimulation

Optogenetic stimulation was performed as described previously [40, 42]. Briefly, the implanted optic fiber was connected to a laser source (B1-465, Inper LLC, Hangzhou, China) by an optical patch cable (200 µm in diameter, N.A. 0.37) through an optical fiber sleeve. A blue laser (465 nm, 5 ms, 30 Hz) with a power of 10 mW was used to activate the PC neurons.

### Fiber photometry recording

Fiber photometry was performed as previously described [43, 44]. AAV-CaMKIIα-GCaMP6m (S0481-9-H5, TaiTool) was injected into the PC. An optical fiber (diameter of 200 uM and 0.37 NA, Inper Tech) was implanted 0.1 mm above the injection sites as described above. Eleven days after implantation, the mice were exposed to CSDS for 10 consecutive days. One day after the completion of CSDS, SIT was performed to screen, and the fluorescence signals of the PC neurons were recorded throughout the SIT using a fiber photometry system (FPS-410/470, Inper) equipped with 470 and 410 nm excitation lasers. The 470 nm that excites fluorescence from GCaMP6m was used to measure neuron activity, and the 410 nm served as a control for movement and bleaching. Photometric data of control and susceptible mice were subjected to autofluorescence background subtraction and imported into MATLAB for further analysis. The calculated (F-F0)/F0 as fluorescence changes (ΔF/F) and heat maps are presented. Post hoc histology was performed to verify the accuracy of viral infection and optic fiber implantation. The data of the mice that deviated from the location were excluded from the analysis.

### Preparation of acute brain slices and electrophysiology

Whole-cell patch-clamp recording on the PC was performed as previously described [42, 44, 45]. We anesthetized and prepared 300 μm coronal slices using a fully automatic vibrating slicer (Leica VT1200S) in ice-cold artificial cerebrospinal fluid (ACSF, composition in mM: 26 NaHCO_3_, 3 KCl, 1.25 NaH2PO4, 10 dextrose, 124 NaCl, 1 MgCl_2_, and 2 CaCl_2_). The slices were recovered for 30 min at 34 °C in ACSF, saturated with 95% O_2_ and 5% CO_2_. The sections were stored at room temperature until further analysis. The acute slices were transferred to a recording chamber perfused with O_2_-saturated ACSF at a rate of 2 ml/min (25 °C). Fluorescent neurons of the PC were visualized using an upright microscope (Nikon Eclipse FN1, Nikon, Tokyo Metropolis, Japan) equipped with an infrared-sensitive CCD camera and ×40 water immersion lens. The recording electrodes (4–6 MΩ) were filled with an internal solution consisting of the following (in mM): 10 KCl, 130 K-gluconate, 130 potassium gluconate, 10 HEPES, 0.5 GTP, 4 ATP, 0.2 EGTA, and 10 Na-phosphocreatine (pH 7.2, 300 mOsm). Electrophysiological signals were acquired using an Integrated Patch-Clamp Amplifier (Sutter Instrument, Novato, CA, USA), digitized at 10 kHz, and Bessel filtered at 2 kHz.

To verify the efficacy of CNO on the chemogenetic-inhibitory virus, we recorded the cells infected with hM4D(Gi) in the current-clamp mode. First, in normal ACSF, a constant current is injected into the cells to record the baseline, followed by continuous recording with ACSF added with 10 μM CNO, which can cause the cells to become hyperpolarized, thereby reducing the excitability of the cells, and finally eluting with normal ACSF and cells returning to their original excitatory state.

To verify the high efficiency of ChR2 activation, in current-clamp mode (holding −70 mV), a pulse of 1 ms blue light from a light-emitting diode source was applied to the acute slice through an optical fiber. The firing of neuron action potentials was recorded under brief light stimulation at 5, 20, 30, and 40 Hz.

### Quantification and statistical analyses

All data analysis was conducted using Prism version 9.2 (GraphPad Software, San Diego, CA, USA). The investigators were blinded to measure different experimental groups. The Shapiro-Wilk W test was used to assess the normality of the data distribution. Two-tailed *t*-tests were used to compare the statistical differences between the two groups; If the normality assumptions could not be accepted, comparisons were made with Wilcoxon rank sum test. One-way analysis of variance (ANOVA) with Bonferroni’s multiple comparison test was used to compare three groups, in which only one factor needed to be considered. If the normality assumptions could not be accepted, comparisons were made with Kruskal–Wallis H. Two-way ANOVA and Tukey’s multiple comparison test were used to analyze the data from the experimental groups with multiple factors. All tests are two-sided. Statistical significance was set at *P* < 0.05.

## Results

### Chronic social defeat stress (CSDS) induces learning and memory impairment and mood-related disorders

**Fig. 1:**
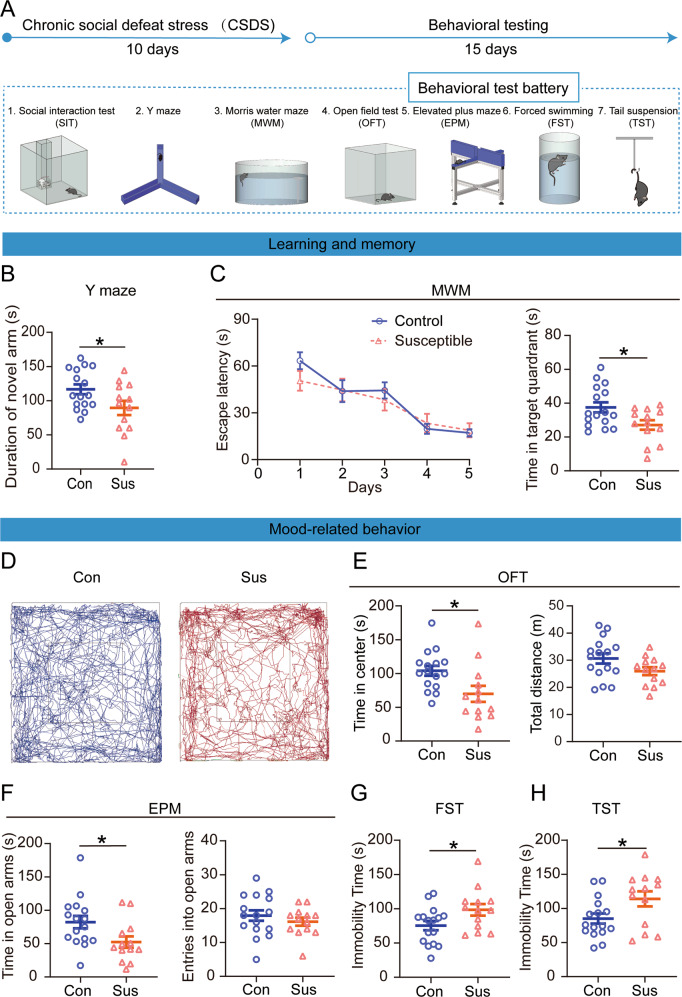
Learning and memory dysfunctions and mood-related disorders in susceptible mice. **A** Experimental schedule of the chronic social defeat stress and behavioral tests. **B** The time in the novel arm was reduced for the susceptible mice (t_27_ = 2.204, *p* = 0.0363; Unpaired student’s *t*-test). Con control, *n* = 16; Sus susceptible, *n* = 13. **C** Escape latency (F _(1, 27)_ = 0.239, *p* = 0.629; Two-way repeated measures ANOVA) and the time spent in the target quadrant (t_27_ = 2.481, *p* = 0.020; Unpaired student’s *t*-test). Con, n = 16; Sus, *n* = 13. **D** Representative animal trace of the open field test in the control and susceptible mice. **E** The susceptible mice spent less time in the center (t_27_ = 2.556, *p* = 0.017; Unpaired student’s *t*-test); However, the total distance was not different between the control and susceptible mice (t_27_ = 1.908, *p* = 0.067; Unpaired student’s *t*-test). Con, *n* = 16; Sus, *n* = 13. **F** The susceptible mice spent less time in the open arms than the control mice (*t*_27_ = 2.309, *p* = 0.029; Unpaired student’s *t*-test). The entries into the open arms did not change in the control and susceptible mice (*t*_27_ = 0.883, *p* = 0.385; Unpaired student’s *t*-test). Con, *n* = 16; Sus, *n* = 13. **G** Results are expressed as the immobility time in the forced swimming test after stress (*t*_27_ = 2.169, *p* = 0.039; Unpaired student’s *t*-test) and **H** the immobility time in the tail suspension test (*t*_27_ = 2.256, *p* = 0.032; Unpaired student’s *t*-test). Con, *n* = 16; Sus, *n* = 13. Data are presented as the mean ± SEM. *p < 0.05.

To investigate whether CSDS exposure impairs learning and memory, we performed the Y-maze and Morris water maze (MWM) tests on mice that experienced CSDS, along with several tests relevant to mood-related disorders (Fig. 1A). Mice were exposed to CSDS for 10 days, and avoidance behavior was tested using the social interaction test (SIT). Control mice show a strong tendency to spend longer or equal amounts of time in the interaction zone in the second session compared with the first session, so their social interaction (SI) ratio >1. After experiencing CSDS, the mice showed social aversion, that is, SI ratio <1, and were classified as susceptible mice, whereas mice with SI ratio >1 as resilient mice. In addition, the susceptible mice showed a reduction in social interaction time and increased time in the corner when a CD1 mouse was present compared to the control and resilient mice (Supplementary Fig. 1). As there were no behavioral differences between the control and resilient mice, only the control and susceptible mice were used for subsequent experiments.

Firstly, Y-maze and MWM tests were used to examine whether CSDS affected learning and memory function in mice. Spatial memory was measured using the Y-maze test. In this test, entering the novel arm more frequently indicated that mice had good spatial memory [46]. The results of the Y-maze test showed that susceptible mice spent less time in the novel arms than the control mice (Fig. 1B). In the acquisition phase of the MWM, there was no significant difference between the latency time of susceptible and control mice to reach the platform (Fig. 1C). One day after the acquisition phase, the platform was removed from the pool for a 90 s probe trial, and the duration in the target quadrant of susceptible mice was much less than that of control mice (Fig. 1C). These results demonstrate that CSDS results in learning and memory dysfunctions.

Consistent with a previous description of CSDS models, susceptible mice displayed significant mood-related disorders [47]. First, it is well known that the reduction in time spent in the center of the open field test (OFT) and time spend in the open arm of the elevated plus maze test (EPM) was correlated with anxiety behaviors in rodents. The OFT showed that the time that the susceptible mice spent in the center area was significantly reduced compared to the control mice, and the total distance between the control and susceptible mice was not significantly different (Fig. 1D, E). In addition, in the EPM, the susceptible mice spent less time in the open arms than the control mice, but entry into the open arms was not significant (Fig. 1F). Finally, the forced swimming test (FST) and tail suspension test (TST) were used to detect the depression-like behavior. Results showed that the immobility time of the susceptible mice was significantly prolonged compared to that of control mice in both FST and TST (Fig. 1G, H). Taken together, these results indicate that susceptible mice exhibit mood-related disorders.

### PC CaMKIIα expressing neurons are preferentially activated by chronic social defeat stress

**Fig. 2:**
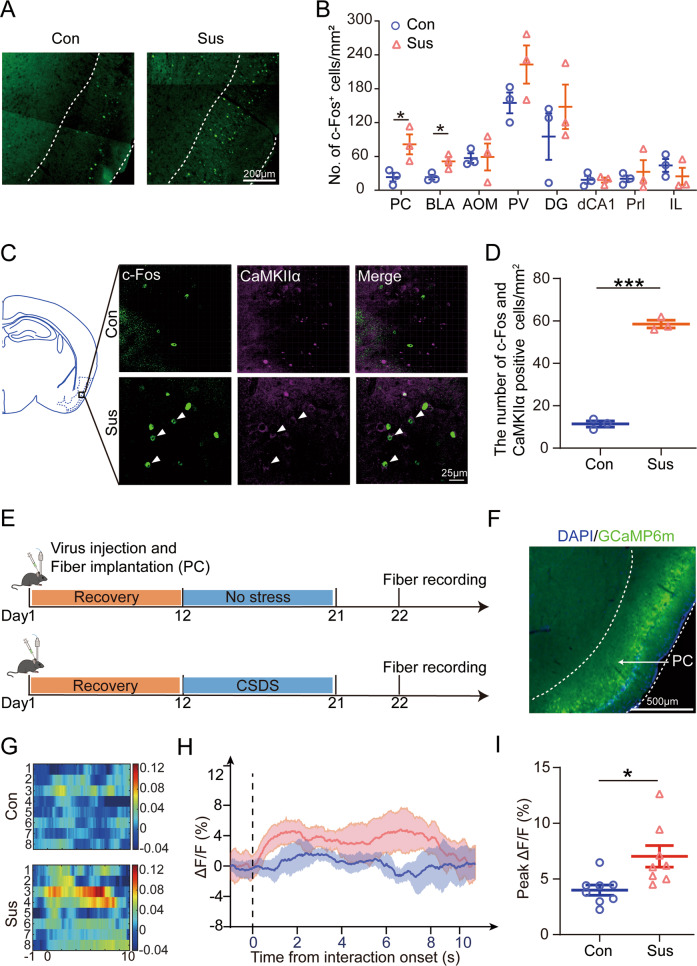
Piriform cortex (PC) excitability neurons are activated by social defeat. **A** After SIT-90 min, mice were sacrificed followed by c-Fos staining to detect the neuronal activity. The c-Fos-positive cells in the PC of the control (left) and susceptible (right) mice were shown through immunofluorescence. Scale bar, 200 μm. **B** The mean density of c-Fos-positive cells in the PC (*t*_4_ = 3.009, *p* = 0.0396; Unpaired student’s *t*-test), BLA (*t*_4_ = 3.273, *p* = 0.0307; Unpaired student’s *t*-test), AOM (*t*_4_ = 0.08456, *p* = 0.9367; Unpaired student’s *t*-test), PV (*t*_4_ = 1.764, *p* = 0.1524; Unpaired student’s *t*-test), DG (*t*_4_ = 0.9232, *p* = 0.4081; Unpaired student’s *t*-test), dCA1 (*t*_4_ = 0.057, *p* = 0.9573; Unpaired student’s *t*-test), Prl (*t*_4_ = 0.5723, *p* = 0.5978; Unpaired student’s *t*-test) and IL (*t*_4_ = 1.019, *p* = 0.3657; Unpaired student’s *t*-test). Con, *n* = 3; Sus, *n* = 3. **C** Merged confocal image of c-Fos (Green) co-stained with CaMKIIα (Purple) in PC slices. The white arrowheads indicate the colocalized cells that expressed both c-Fos and CaMKIIα neurons. Scale bar, 25 μm. **D** The density of c-Fos and CaMKIIα co-labeled cells were measured (*t*_4_ = 20.36, *p* < 0.001; Unpaired student’s *t*-test). Con, *n* = 3; Sus, *n* = 3. **E** Experimental timeline for recording dynamic Ca^2+^ signals in GCaMP6m-expressing PC neurons. **F** Representative images of the injection site in the PC. Scale bar, 500 μm. **G** Heatmap showing Ca^2+^ signals aligned to the onset of social interaction in the social interaction. Each row represents a trial. **H** Representative line of the averaged Ca^2+^ signals. **I** Summary plots of the ΔF/F signal (*t*_14_ = 2.846, *p* = 0.0130; Unpaired student’s *t*-test). Con, *n* = 8, mice = 3; Sus, *n* = 8, mice = 3. Data are presented as the mean ± SEM. *p < 0.05, ***p < 0.001.

It has been reported that c-Fos was a reliable marker for detecting neuronal activation induced by various behavioral stimuli [48]. To clarify the specific area that responds to CSDS and induces learning and memory dysfunction, the brain slices of the control and susceptible mice were fluorescently labeled with c-Fos after SIT. We counted cell density in various brain regions, and only brain regions which rich in c-Fos expression and have been reported to be involved in learning and memory were displayed, including PC, basolateral amygdaloid nucleus (BLA), anterior olfactory area (AOM), paraventricular thalamic nucleus (PV), dentate gyrus (DG), dorsal hippocampus CA1 (dCA1), prelimbic cortex (Prl), and infralimbic cortex (IL) [49–52]. Results showed that the density of c-Fos positive cells in the PC and BLA of the susceptible mice was significantly higher than in the control mice, whereas there was no significant difference in AOM, PV, DG, dCA1, Prl, and IL. It is noteworthy that the difference of c-Fos expression in the PC was most significant after CSDS treatment (Fig. 2A, B and Supplementary Fig. 2A). However, immunohistochemistry results showed that c-Fos expression in PC did was not different between the control group and mice exposed to acute social defeat (ASD) (Supplementary Fig. 3).

It is known that PC mainly consists of excitatory cells that can be immunolabeled by CaMKIIα antibody (CaMKIIα^+^) and contains small amounts of inhibitory GABAergic interneurons that are CaMKIIα immune negative cells (CaMKIIα^−^) [19]. To determine which type of neurons in the PC were involved in the learning and memory dysfunction of susceptible mice, double-labeled immunofluorescence staining of CaMKIIα and c-Fos was performed on brain slices of susceptible mice and control mice (Fig. 2C). In the PC of control mice, a small number of c-Fos^+^ and were CaMKIIα^+^ co-labeled neurons were found. After CSDS treatment, the density and ratio of CaMKIIα^+^ and c-Fos^+^ co-labeled cells increased significantly, and these double-labeled neurons are distributed in layers I–III of PC, with the most in layer II and less in layers I and III (Fig. 2D and Supplementary Fig. 4A). These results indicate that CaMKIIα expressing neurons in the PC were selectively activated by CSDS exposure.

To further confirm the response of PC CaMKIIα expressing neurons to CSDS, dynamic calcium signals during SIT were recorded using in vivo fiber photometry (Fig. 2E). AAV2/9-CaMKIIα-GCaMP6m was delivered to the PC by stereotaxic injection to express the Ca^2+^ indicator GCaMP6m in PC CaMKIIα expressing neurons (Fig. 2F and Supplementary Fig. 4B), and then an optical fiber was implanted into the injection site. The GCaMP6m fluorescence was monitored during the SIT after 10 days of CSDS. We found that calcium signals of PC neurons increased slightly within seconds of interaction onset but increased more in susceptible mice than in control mice (Fig. 2G–I). These results suggest that PC CaMKIIα expressing neurons are preferentially overactivated after CSDS exposure.

To further clarify the relationship between PC hyperexcitability and learning and memory impairment, we detected the expression of c-Fos in the PC after MWM test. Results showed that the c-Fos immunoactivity neurons in the PC of susceptible mice were also significantly increased after MWM compared with the control group (Supplementary Fig. 4C, D). Although cognitive impairment has been reported in chronic restraint stress (CRS) and chronic unpredictable mild stress (CUMS) mice, our results showed that the c-Fos immunoactivity neurons were not increased after MWM in PC of CUMS and CRS mice (Supplementary Fig. 5). Therefore, it is speculated that the overactivation of CaMKIIα expressing neurons in the PC may be unique to CSDS-induced learning and memory impairment.

### Chemogenetic activation of PC CaMKIIα expressing neurons induced learning and memory dysfunction but not mood-related disorders in mice

**Fig. 3:**
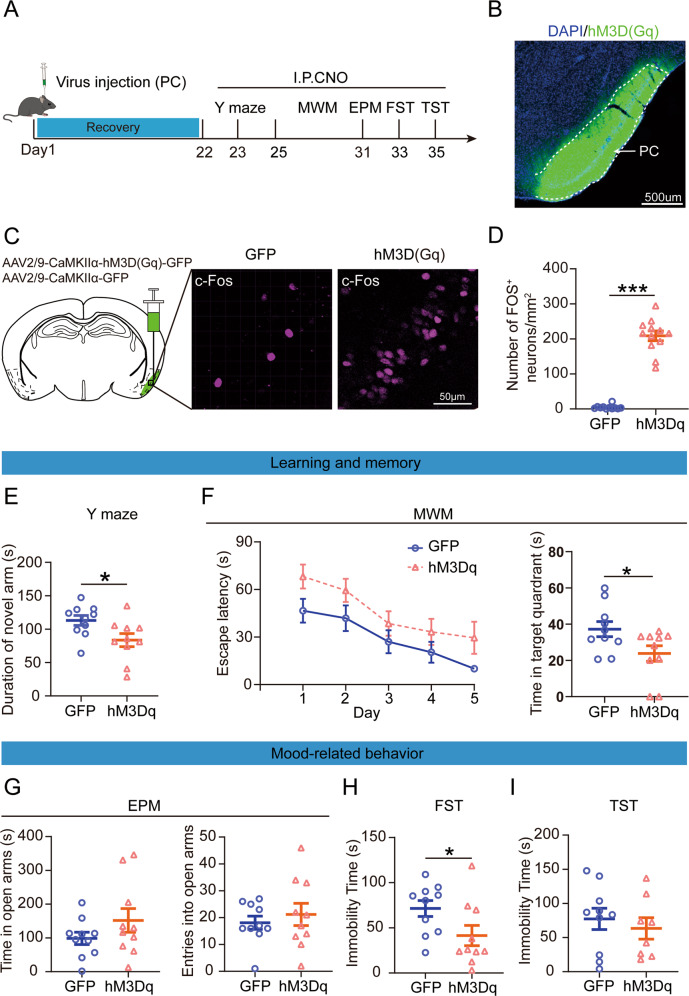
Effects of chemogenetic activate the piriform cortex (PC) excitability neurons-induced learning and memory dysfunction but not mood-related disorders. **A** Experimental timeline of virus injection for investigating the behavioral impact, CNO injections (3.3 mg/kg). **B** Schematic of the sagittal section of the mouse brain shows hM3D(Gq) virus injection. **C** The c-Fos positive cells were activated after CNO injection. Scale bar, 50 μm. **D** Mean count of the c-Fos-positive neurons in the PC following CNO injection (*z* = −4.066, *p* < 0.001; Wilcoxon rank sum test). GFP, injection with GFP-expressing viral vector in mice, *n* = 11, mice = 3; hM3Dq, injection with hM3D(Gq)-expressing viral vector in mice, *n* = 12, mice = 3. **E** Quantification of the time spent in the novel arm after injected CNO in hM3Dq and GFP mice (*t*_18_ = 2.390, *p* = 0.028; Unpaired student’s *t*-test). GFP, *n* = 10; hM3Dq, *n* = 10. **F** Escape latency was not significantly different between hM3Dq and GFP mice (*F*_(1, 18)_ = 4.204, *p* = 0.055; Two-way repeated measures ANOVA), but hM3Dq mice spent less time in the target quadrant than GFP mice (*t*_18_ = 2.183, *p* = 0.0425; Unpaired student’s *t*-test). GFP, *n* = 10; hM3Dq, *n* = 10. **G** Time in the open arms (*t*_18_ = −1.341, *p* = 0.197; Unpaired student’s *t*-test) and open arm entries (*t*_18_ = −0.644, *p* = 0.528; Unpaired student’s *t*-test) during the elevated plus maze. GFP, *n* = 10; hM3Dq, *n* = 10. **H** The immobility time in the forced swimming test (*t*_18_ = 2.101, *p* = 0.0499; Unpaired student’s *t*-test. GFP, *n* = 10; hM3Dq, *n* = 10) and **I** tail suspension test (*t*_16_ = 0.6192, *p* = 0.545; Unpaired student’s *t*-test. GFP, *n* = 10; hM3Dq, *n* = 8). Data are presented as the mean ± SEM. *p < 0.05, ***p < 0.001.

To test whether the overactivation of PC CaMKIIα expressing neurons contributed to learning and memory dysfunction, we selectively activated these neurons using chemogenetic methods (Fig. 3A). A recombinant AAV reporter virus expressing hM3D(Gq) or GFP, controlled by the CaMKIIα promotor (AAV2/9-CaMKIIα-hM3D(Gq)-GFP or AAV2/9-CaMKIIα-GFP), was unilaterally delivered into the PC by stereotactic injection (Fig. 3B). Three weeks after virus injection, Clozapine N-oxide (CNO) was intraperitoneally injected 45 min before the test to selectively activate the PC CaMKIIα expressing neurons. To ensure the effectiveness of the virus, c-Fos was immunolabeled on brain slices, and much more c-Fos^+^ cells were observed in the PC cortex of hM3D(Gq)-injected mice than in GFP-injected mice (Fig. 3C, D).

First, we tested the effect of active PC CaMKIIα expressing neurons on learning and memory. In the Y-maze, the time spent by hM3D(Gq)-injected mice in the novel arm was significantly shorter than that in GFP-injected mice, indicating that spatial memory was damaged by PC excitatory activation (Fig. 3E). In the MWM test, the hM3D(Gq) mice had no significant difference to find the platform in the first 5 days of training. On the sixth day, the hM3D(Gq)-injected mice spent less time in the target quadrant (Fig. 3F). These results indicate that activation of PC CaMKIIα expressing neurons is closely related to learning and memory impairment.

Finally, we evaluated the effect of PC activation on mood-related behaviors using EPM, FST, and TST. The results showed that hM3D(Gq)-injected mice did not change the time length and number of entries in the open arms of the EPM test compared with GFP-injected mice (Fig. 3G). In addition, chemcogenetically active PC CaMKIIα expressing neurons did not change the immobility time in the TST but shortened the immobility time in the FST (Fig. 3H, I). These results indicate that active PC CaMKIIα expressing neurons do not induce mood-related disorders.

### Optogenetic activation of PC CaMKIIα expressing neurons induced learning and memory dysfunction but not mood-related disorders in mice

**Fig. 4:**
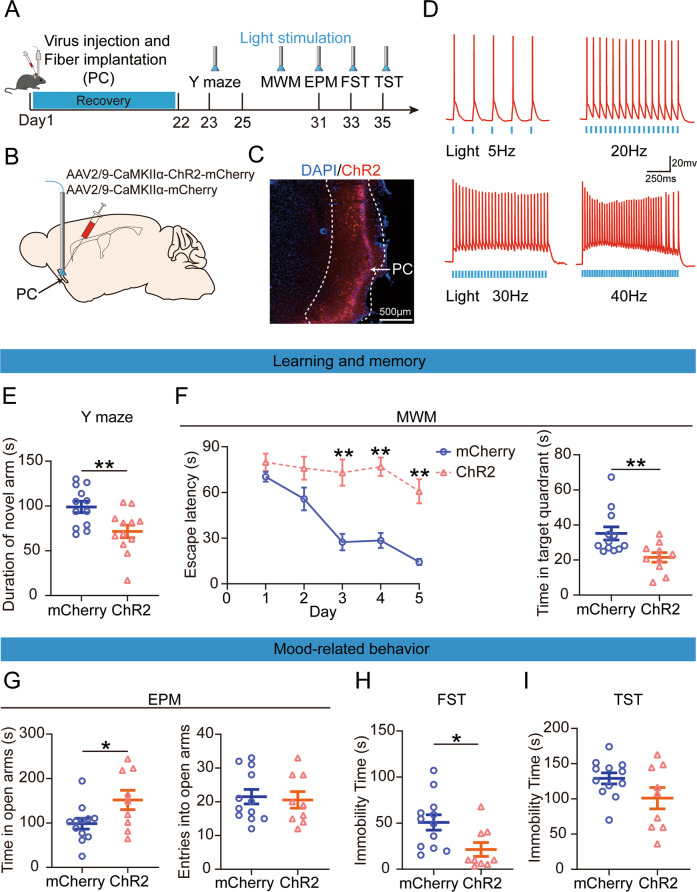
Optogenetic activation of the piriform cortex (PC) excitability neurons decrease learning and memory but not mood-related behavior. **A** Experimental timeline for virus injection for investigating the behavioral impact of optogenetic activation. Blue light pulses at 30 Hz, 5 ms,10 mW. **B**, **C** Schematic of the sagittal section of the mouse brain shows ChR2 virus injection. **D** Representative recording of the action potential firing of CaMKIIα expressing neurons in response to 5, 20, 30, and 40 Hz light photostimulation. **E** Quantification of the time spent in the novel arm by light photostimulation (*t*_22_ = 2.886, *p* = 0.009; Unpaired student’s *t*-test). mCherry, injection with mCherry-expressing viral vector in mice, *n* = 12; ChR2, injection with ChR2-expressing viral vector in mice, *n* = 12. **F** Escape latency (*F*_(1, 20)_ = 21.269, *p* < 0.001; Two-way repeated measures ANOVA) and the time spent in the target quadrant (*z* = −2.672, *p* = 0.008; Wilcoxon rank sum test). mCherry, *n* = 12; ChR2, *n* = 10. **G** Time in the open arms (*t*_19_ = −2.291, *p* = 0.034; Unpaired student’s *t*-test) and open arm entries (*t*_19_ = 0.288, *p* = 0.777; Unpaired student’s *t*-test) during the elevated plus maze. mCherry, *n* = 12; ChR2, *n* = 9. **H** The immobility time in the forced swimming test (*z* = −2.417, *p* = 0.016; Wilcoxon rank sum test) and **I** tail suspension test (*t*_19_ = 1.768, *p* = 0.093; Unpaired student’s *t*-test). mCherry, *n* = 12; ChR2, *n* = 9. Data are presented as the mean ± SEM. *p < 0.05; **p < 0.01.

To control neuronal activity more precisely on a millisecond scale, we next used optogenetic methods to reveal the effects of PC neuronal excitation on animal behavior (Fig. 4A). We stereotaxically injected AAV2/9-CaMKII-ChR2-mCherry or AAV2/9-CaMKIIα-mCherry into the PCs (Fig. 4B). The expression of the viral vector was confirmed by fluorescence microscopy (Fig. 4C). To verify the function of ChR2, in vitro whole-cell recording was performed. Results indicated that precise and reliable firing could be induced in ChR2-expression neurons by blue light up to frequencies of 30 Hz (Fig. 4D). In addition, 30 Hz blue light could induce much more c-Fos expression in ChR2-expression neurons than mCheery expression neurons (Supplementary Fig. 6).

Blue light optical stimulation (5 ms, 30 Hz, 10 mW/mm²) was applied during the behavioral test. Once the blue light was turned on, the mice experienced convulsions, but the phenomena disappeared 10 min later, and all behavioral tests were performed after the phenomena disappeared. Optical stimulation during the Y-maze test significantly decreased the duration of ChR2-expression mice in the novel arm compared to mCherry expression mice (Fig. 4E). In the spatial acquisition phase of the MWM test, ChR2-expression mice needed more time to find the platform than mCherry expression mice. In the probe trial, ChR2-expression mice spent less time in the target quadrant (Fig. 4F).

We then evaluated mood-related disorders using the EPM, FST, and TST. Optogenetic activation of PC significantly increased the length of time spent in the open arms but did not change the number of mice entering the open arm (Fig. 4G). Additionally, immobility time in the FST was significantly reduced by optogenetic stimulation in ChR2-expression mice compared with mCherry-expressing mice, whereas the immobility time in the TST was not significantly affected (Fig. 4H, I).

A comprehensive analysis of the above results demonstrated that the optogenetic and chemogenomic activation of PC CaMKIIα expressing neurons induces learning and memory disorders. In contrast, hyperexcitation of the PC did not cause mood-related disorders.

### Chemogenetic inhibition of CaMKIIα expressing neurons in the PC cortex alleviates learning and memory dysfunction but not mood-related disorders induced by CSDS

**Fig. 5:**
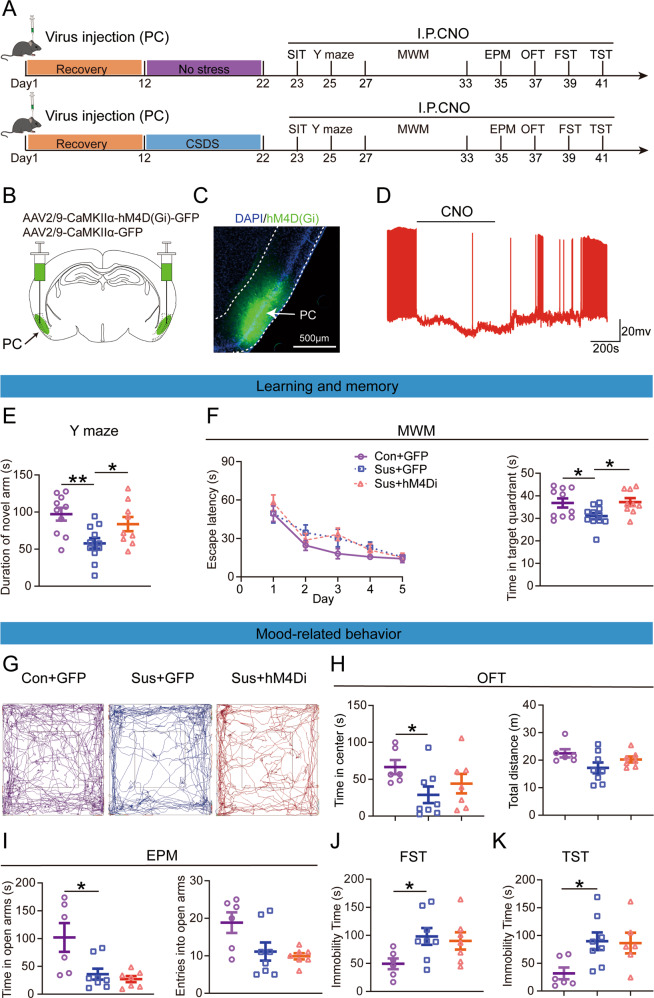
Learning and memory dysfunction but not mood-related disorders were alleviated by the chemogenetic inhibition of the piriform cortex (PC) excitability neurons. **A** Experimental timeline of virus injection for investigating the behavioral impact, CNO injections (3.3 mg/kg). **B**, **C** Schematic of the sagittal section of the mouse brain shows hM4D(Gi)-GFP virus injection. **D** Current-clamp recording of expressing hM4D(Gi) virus in the PC. The representative trace shows that PC neurons expressing hM4D(Gi) can be inhibited by bath application of 10 μM CNO. **E** The time spent in the novel arm following PC injections of AAV-GFP and AAV-hM4D(Gi) (F _(2, 27)_ = 5.996, Con + GFP vs Sus + GFP: *p* = 0.002, Sus + GFP vs Sus + hM4Di: *p* = 0.035; One-way ANOVA). Con + GFP, injection with GFP-expressing viral vector in control mice, *n* = 10; Sus + GFP, injection with GFP-expressing viral vector in susceptible mice, *n* = 11; Sus + hM4Di, injection with hM4D(Gi)-expressing viral vector in susceptible mice, *n* = 9. **F** Escape latency (*F*_(2, 27)_ = 1.878, *p* = 0.172; Two-way repeated measures ANOVA) and the time spent in the target quadrant (F _(2, 27)_ = 4.209, Con + GFP vs Sus + GFP: *p* = 0.0402, Sus + GFP vs Sus + hM4Di: *p* = 0.0334; One-way ANOVA). Con + GFP, *n* = 10; Sus + GFP, *n* = 11; Sus + hM4Di, *n* = 9. **G** Representative animal trace of the open field test. **H** The time in the center (F _(2,18)_ = 3.553, Con + GFP vs Sus + GFP: *p* = 0.0299, Sus + GFP vs Sus + hM4Di: *p* = 0.3182; One-way ANOVA) and the total distance did not change after the injection of CNO (F _(2,18)_ = 3.696, Con + GFP vs Sus + GFP: *p* = 0.0631, Sus + GFP vs Sus + hM4Di: *p* = 0.3122; One-way ANOVA). Con + GFP, *n* = 6; Sus + GFP, *n* = 8; Sus + hM4Di, *n* = 7. **I** The hM4Di-susceptible mice presented no change in terms of both open arm exploration (x^2^ = 7.427, Con + GFP vs Sus + GFP: *p* = 0.0317, Sus + GFP vs Sus + hM4Di: *p* > 0.999; Kruskal–Wallis H) and entries into the open arm (F _(2,18)_ = 4.654, Con + GFP vs Sus + GFP: *p* = 0.167, Sus + GFP vs Sus + hM4Di: *p* = 0.950; One-way ANOVA) compared to GFP-susceptible mice in the EPM test. Con + GFP, *n* = 6; Sus + GFP, *n* = 8; Sus + hM4Di, *n* = 7. **J** The immobility time in the forced swimming test (F _(2, 18)_ = 3.186, Con + GFP vs Sus + GFP: *p* = 0.0494, Sus + GFP vs Sus + hM4Di: *p* = 0.8871; One-way ANOVA) and **K** tail suspension test (F _(2,17)_ = 4.113, Con + GFP vs Sus + GFP: *p* = 0.0314, Sus + GFP vs Sus + hM4Di: *p* = 0.9842; One-way ANOVA). Con + GFP, *n* = 6, Sus + GFP, *n* = 8, Sus + hM4Di, *n* = 6. Data are presented as the mean ± SEM. *p < 0.05; **p < 0.01.

As active PC was found to induce learning and memory impairment, we examined whether inhibiting PC CaMKIIα expressing neurons could alleviate CSDS-induced learning and memory dysfunction. Next, we inhibited PC CaMKIIα expressing neurons during behavior testing using the chemogenetic method (Fig. 5A). We injected AAV2/9-CaMKIIα-hM4D(Gi)-GFP or AAV2/9-CaMKIIα-GFP into the PC (Fig. 5B, C). To confirm the function of hM4D(Gi), neuronal activity was recorded in acute brain slices three weeks after injection. Consistent with the expected results, the firing frequency of hM4D(Gi)-expression neurons significantly decreased after CNO application (Fig. 5D).

After exposure to CSDS, the control and susceptible mice were selected for subsequent experiments (Supplementary Fig. 7). The Y-maze test and MWM were used to examine the effect of inhibiting PC CaMKIIα expressing neurons on learning and memory function. CNO (3.3 mg/kg) was administered intraperitoneally 45 min before behavioral testing. The Y-maze results showed that GFP-expression susceptible mice spent less time in novel arms than control mice, but hM4D(Gi)-expression significantly prolonged the time spent in novel arms compared to GFP-expression susceptible mice (Fig. 5E). Moreover, in MWM, CNO administration did not change escape latency in the spatial acquisition phase but significantly increased the duration in the target quadrants (Fig. 5F).

We also tested whether inhibition of PC activation could relieve CSDS-induced mood-related disorders. Compared with the GFP-expressing susceptible mice, hM4D(Gi)-expression did not affect the duration in the center, the total traveled distance in the OFT (Fig. 5G, H), the duration and entries in the EPM (Fig. 5I), and the immobility length in FST and TST (Fig. 5J, K).

These results indicate that hyperexcitation of PC CaMKIIα expressing neurons is essential for learning and memory dysfunction but is not involved in mood-related disorders induced by CSDS.

## Discussion

We investigated whether PC is involved in the learning and memory dysfunction induced by CSDS. We found that CSDS-induced both mood-related disorders and learning and memory dysfunction in mice, accompanied by the overactivation of PC CaMKIIα expressing neurons. We also demonstrated that activation of PC CaMKIIα expressing neurons by optogenetic and chemogenetic methods induced learning and memory dysfunction, but not mood-related disorders. Furthermore, inhibition of PC CaMKIIα expressing neurons alleviated learning and memory impairment, but not mood-related disorders induced by CSDS.

Mood-related disorders are one of the most common mental illnesses, including depressed-like behavior and anxiety [53]. Learning and memory deficits have been identified in patients with depression, including dysfunction of executive function, working memory, emotional memory, episodic memory, and semantic memory [47]. Repeated exposure to social defeat stress results in a robust mood-related phenotype in rodents [9]. However, the relationship between social defeat and learning and memory dysfunction remains controversial [54]. This study confirmed that mice would induce mood-related symptoms as indicated by the OFT, EPM, FST, and TST. In addition, our results showed that after exposure to CSDS for 10 days, mice spent less time in the novel arm of the Y-maze test, which is a widely used indicator of working memory and spatial memory impairment. Even though the Y-maze was based on spatial novelty, the novelty preference should be measured with a short (2 min) intertrial interval between the first trial and the second trial, while the memory was examined with longer intervals (30 min, 1 h, and 2 h). Thus, our Y-maze results mainly reflect learning and memory deficits [46]. This finding is consistent with those of previous studies in which chronic stress decreased the spontaneous alternation rate in the T-maze test [47]. In addition, we demonstrated that CSDS also damaged reference learning and memory in the MWM test. This result was in accordance with that of rats or mice subjected to chronic unpredictable [55], variable, mild stress, but in contrast to a study in which the MWM was performed on the 14th day after social defeat exposure [56]. The discrepancy may be due to the that the learning and memory impairment may decrease over time after the cessation of social defeat and become undetectable.

Animal studies showed that chronic stress causes structural remodeling in the brain structures, including the prefrontal cortex and the hippocampus, such as dendritic shortening and spine loss, neuronal atrophy, and the inhibition of neurogenesis, which alter the function of glutamate receptors, which result in cognitive impairments [57–59]. Chronic restraint stress impaired long-term potentiation (LTP) in the ventral subiculum-nucleus accumbens (NAc) pathway, induced structural and functional alterations in Medial prefrontal cortex (mPFC), and impaired the hippocampal-dependent spatial memory [59]. CUMS-induced learning and memory impairment was triggered by an inflammatory response in the rat hippocampus, which results in oxidative stress injury and impacts the synaptic plasticity of hippocampal neurons. However, we found that the activation of mPFC and hippocampus did not change obviously, whereas the PC CaMKIIα expressing neurons were selectively activated after exposure to CSDS. But, PC was not active by ASD, CRS, and CUMS stress. This may be due to the fact that C57BL/6 J mice and CD1 live in the same cage during CSDS and receive olfactory stimulation from CD1 for a long time in addition to psychological and physical stimulation. Therefore, compared with other types of stress, CSDS specifically activated the PC, which is involved in olfactory information processing. PC belongs to the limbic system and is vulnerable to excitotoxicity. PC overactivation plays an important role in the epilepsy circuit of rodents and has highly epileptogenic properties [60]. Thus, the mice triggered motor seizures after optogenetically active PC in this study, which was in accordance with the phenotype of unilateral microinjection of picomolar amounts of glutamate receptor agonists into the PC of rats and non-human primates [61]. The motor abnormality may be due to photogenetic activation causing the neurons of the PC to fire synchronously. Although chemical activation induced neurons to be more excitatory, the firing of neurons was not synchronous. Therefore, the symptoms of motor seizures were much less severe. Electroconvulsive therapy (ECT) is a highly effective treatment for severe treatment-refractory depression [62]. In ECT, when the pulsed electrical current passes between two electrodes on the head, selective brain regions, such as PC were activated, and activity-dependent genes were expressed, and then elicit by brief tonic-clonic seizures [63, 64]. In addition, adverse effects such as cognitive and memory deficits have been reported after ECT [65, 66]. Therefore, combined with our experimental results, it is speculated that hyperactivation of the PC may be the overlapping mechanism for ECT-induced epileptic seizures and learning and memory deficit.

In addition, PC is also an important structure of the olfactory system and receives direct projections from the olfactory bulb via the lateral olfactory tract. Overactivation of PC is an important cause of olfactory dysfunction in PD patients [67]. Besides, amyloid-β (Aβ) aggregation-induced PC dysfunction and synaptic plasticity damage have a profound influence on the olfactory function of AD Patients [68, 69]. As the largest part of the olfactory cortex, PC plays an important role in olfactory processing and flavor recognition memory [70]. Olfactory learning could result in increased neuronal excitability, increased synaptic transmission, and long-term potentiation (LTP) like-phenomenon appearing in PC [71]. Here, we inhibited the CaMKIIα expressing neurons of the PC to determine whether PC CaMKIIα expressing neurons were necessary for CSDS-induced learning and memory dysfunction. Although PC has been reported to be associated with mood-related behavior [72], inhibition of the PC CaMKIIα expressing neurons of susceptible mice only alleviated learning and memory impairment but did not alleviate mood-related disorders. We hypothesized that CSDS only activates a group of neurons that are involved in learning and memory function, while the neurons controlling mood-related behaviors were not activated, so chemical inhibition of PC CaMKIIα expressing neurons only alleviated learning and memory impairment in susceptible mice.

**Fig. 6:**
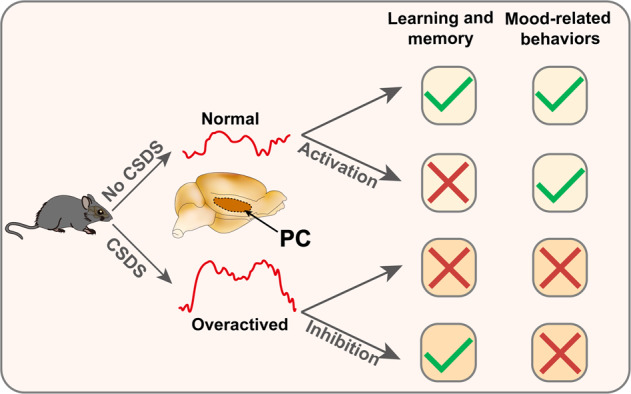
Schematic diagram for PC overactivation mediates CSDS-induced learning and memory behaviors but not mood-related behavior. After mice experienced chronic social defeat stress, the activity of PC-calcium signaling was increased, resulting in learning and memory impairment and mood-related disorders. Then, activation of PC CaMKIIα expressing neurons results in learning and memory dysfunction in mice, but not mood-related disorders. Inhibition of PC CaMKIIα expressing neurons in susceptible mice alleviated learning and memory impairment but had no significant effect on mood-related behavior.

In conclusion, our study adopted chemogenetic and optogenetic methods to enhance PC activation and found that mice showed obvious learning and memory dysfunction without mood-related disorders, suggesting that PC hyperactivation induces learning and memory dysfunction. Inhibition of PC activation in susceptible mice alleviates learning and memory dysfunction but had no significant effect on mood-related behavior. Therefore, our present findings provide evidence that PC plays an important role in learning and memory dysfunction in mice exposed to CSDS, rather than being associated with depression behavior (Fig. 6). These results reveal the underlying mechanism and provide a potential therapeutic target for social defeat-induced learning and memory impairment. However, the mechanism by which PC is activated by CSDS remains to be further studied.

## Supplementary information

SUPPLEMENTAL MATERIAL
