# Supplementary material for: Social defeat drives hyperexcitation of the piriform cortex to induce learning and memory impairment but not mood-related disorders in mice
Source: Transl Psychiatry. 2022 Sep 10;12:380. doi: 10.1038/s41398-022-02151-1 (PMC9464232; doi:10.1038/s41398-022-02151-1)
Supplement: Supplementary file 1 — SUPPLEMENTAL MATERIAL [file 41398_2022_2151_MOESM1_ESM.docx]

**Supplementary information**

**
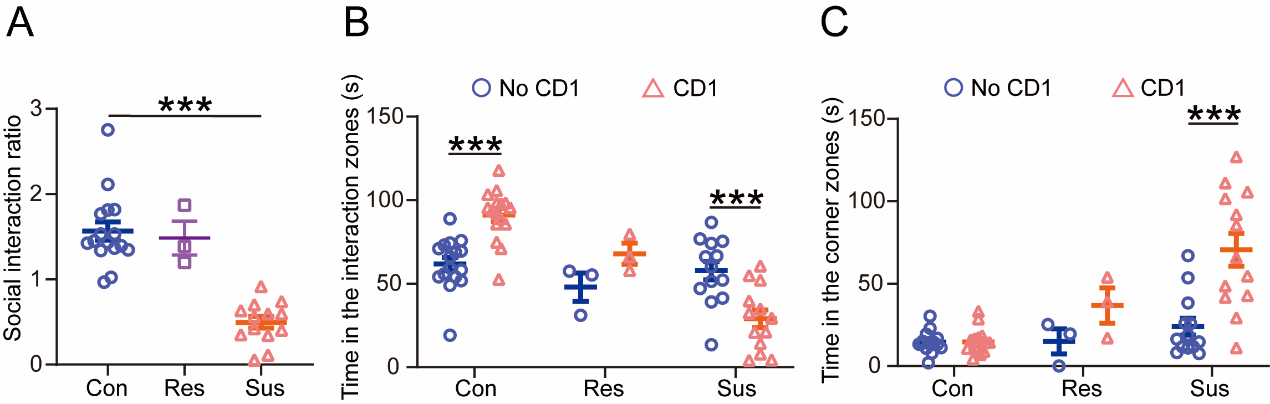
**

**Supplementary Figure 1. CSDS induces avoidance behavior in susceptible mice.**

**(A)** The graph depicts the interaction ratio for the control, resilient, and susceptible mice (*x^2^* = 22.50, Con vs Res: *p* > 0.999; Con vs Sus: *p* < 0.001; Kruskal-Wallis H). Con, control, n = 16; Res, resilient, n = 3; Sus, susceptible, n = 13. **(B)** Column of the time spent in the interaction zone for the control (Interaction: *F* _(2,58)_ = 21.36, *p* < 0.001), resilient (*p* = 0.4703), and susceptible mice (*p* < 0.001). Con, n = 16; Res, n = 3, Sus, n = 13; Two-way ANOVA. **(C)** Time spent in the corner zone for the control (Interaction: *F* _(2,58)_ = 10.26, *p* > 0.999), resilient (*p* = 0.5226), and susceptible mice (*p* < 0.001). Con, n = 16; Res, n = 3, Sus, n = 13; Two-way ANOVA. Data are presented as the mean ± SEM. ****p* < 0.001.


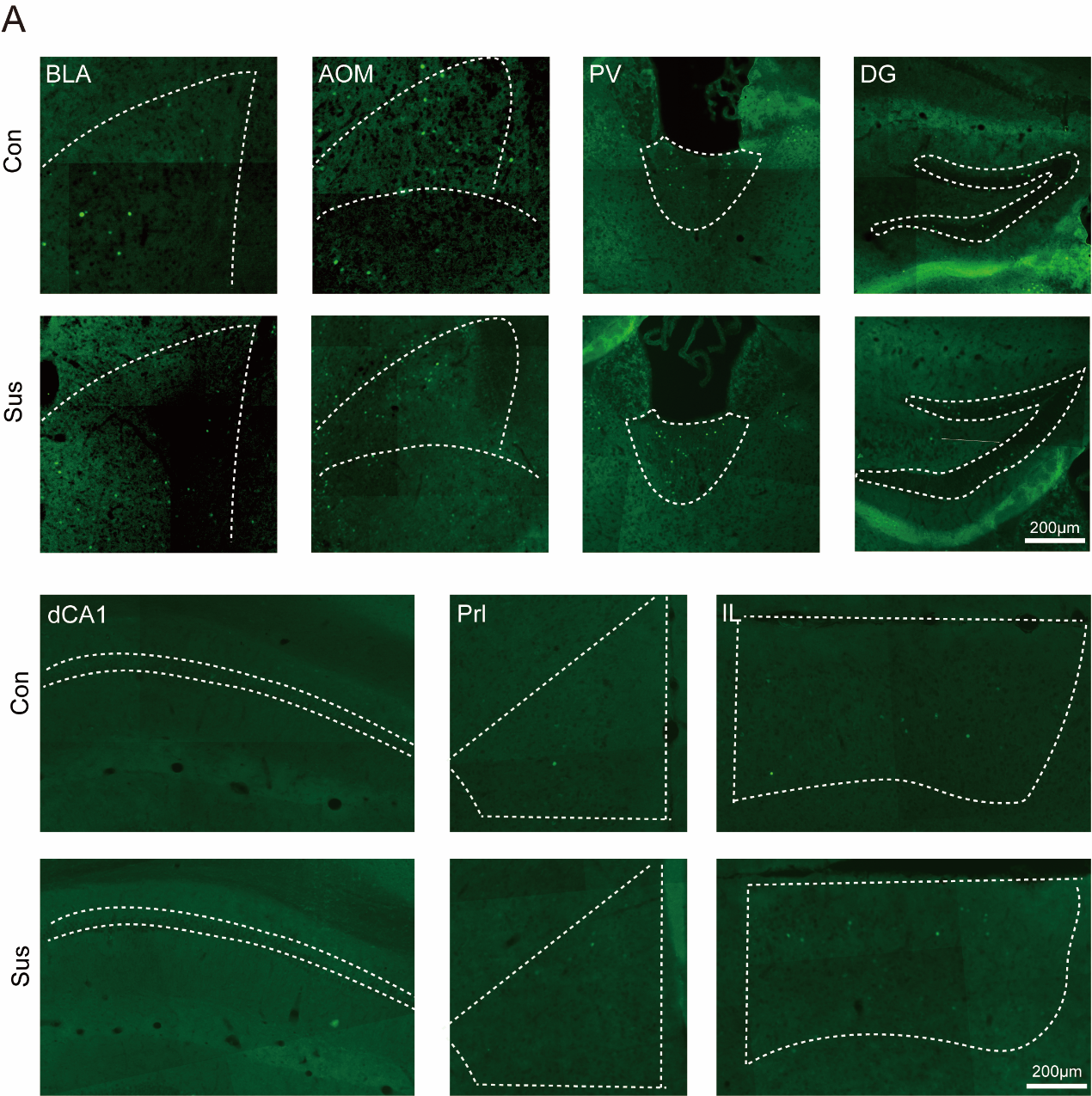


**Supplementary Figure 2. Immunofluorescence images of c-Fos after chronic social defeat stress exposure.**

**(A)** c-Fos positive cells are shown through immunofluorescence images in the BLA, AOM, PV, DG, dCA1, Prl and IL of control (top) and susceptible (bottom) mice. Scale bar, 200 μm.


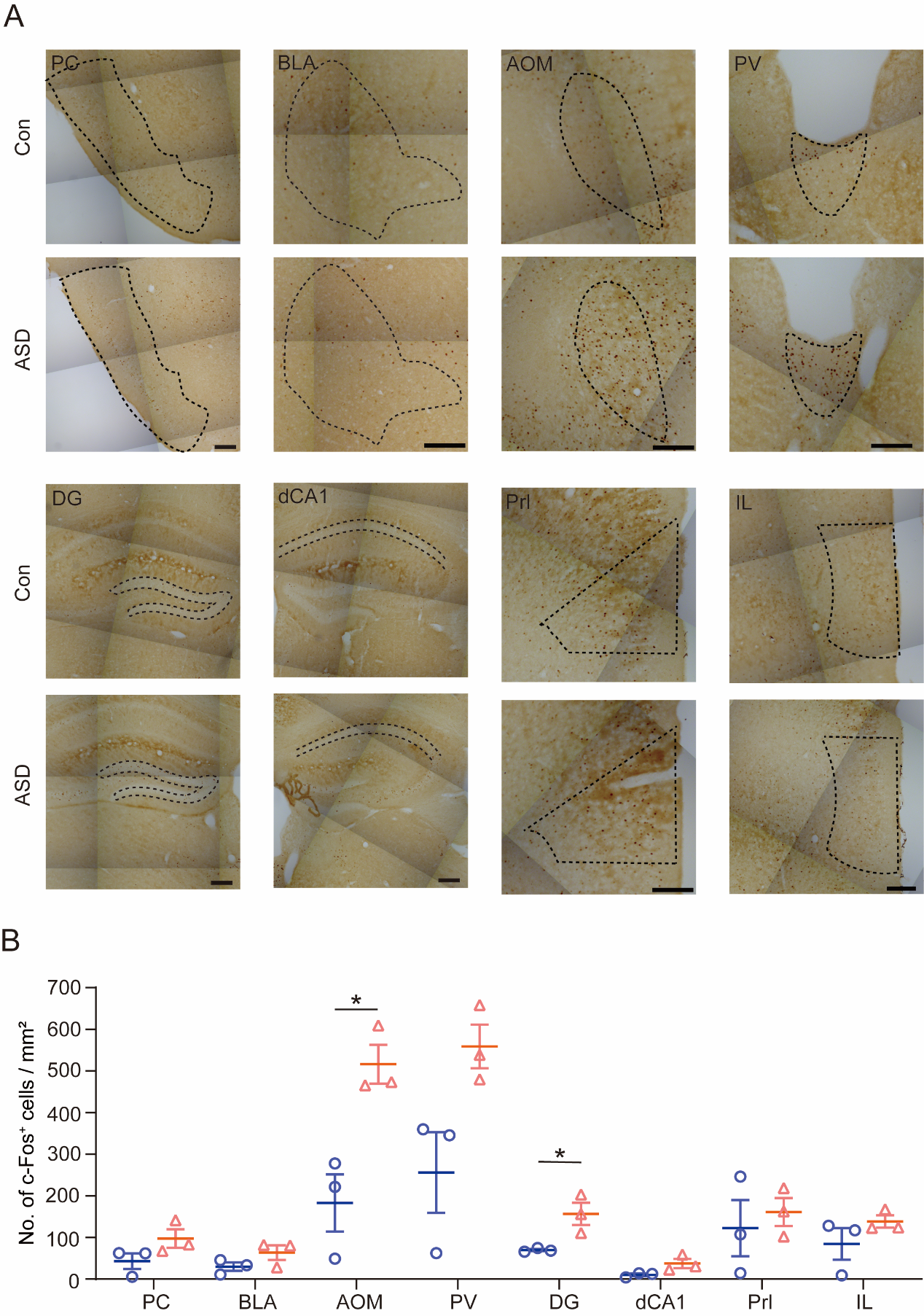


**Supplementary Figure 3. Immunohistochemistry of c-Fos after exposed to acute social defeat stress.**

**(A)** Ninety minutes after ASD, mice were sacrificed to detect c-Fos expression by immunohistochemistry staining. The c-Fos positive cells of control (above) and ASD (below) mice were shown. Scale bar, 200 μm. **(B)** The mean density of c-Fos positive cells in the PC (*z* = -1.964, *p* = 0.1; Wilcoxon rank sum test), BLA (*z* = -1.091, *p* = 0.4; Wilcoxon rank sum test), AOM (*t_4_* = 4.006, *p* = 0.0161; Unpaired student’s t-test ), PV (*t*_4_ = 2.749, *p* = 0.051; Unpaired Student’s t-test), DG (*t*_4_ = -3.243, *p* = 0.032; Unpaired Student’s t-test), dCA1 (*t*_4_ = -2.395, *p* = 0.075; Unpaired Student’s t-test), Prl (*t*_4_ = -0.154, *p* = 0.634; Unpaired Student’s t-test) and IL (*z* = -1.091, *p* = 0.4; Wilcoxon rank sum test). Con, control, n = 3; ASD, acute social defeat stress, n = 3. Data are presented as the mean ± SEM. **p* < 0.05.


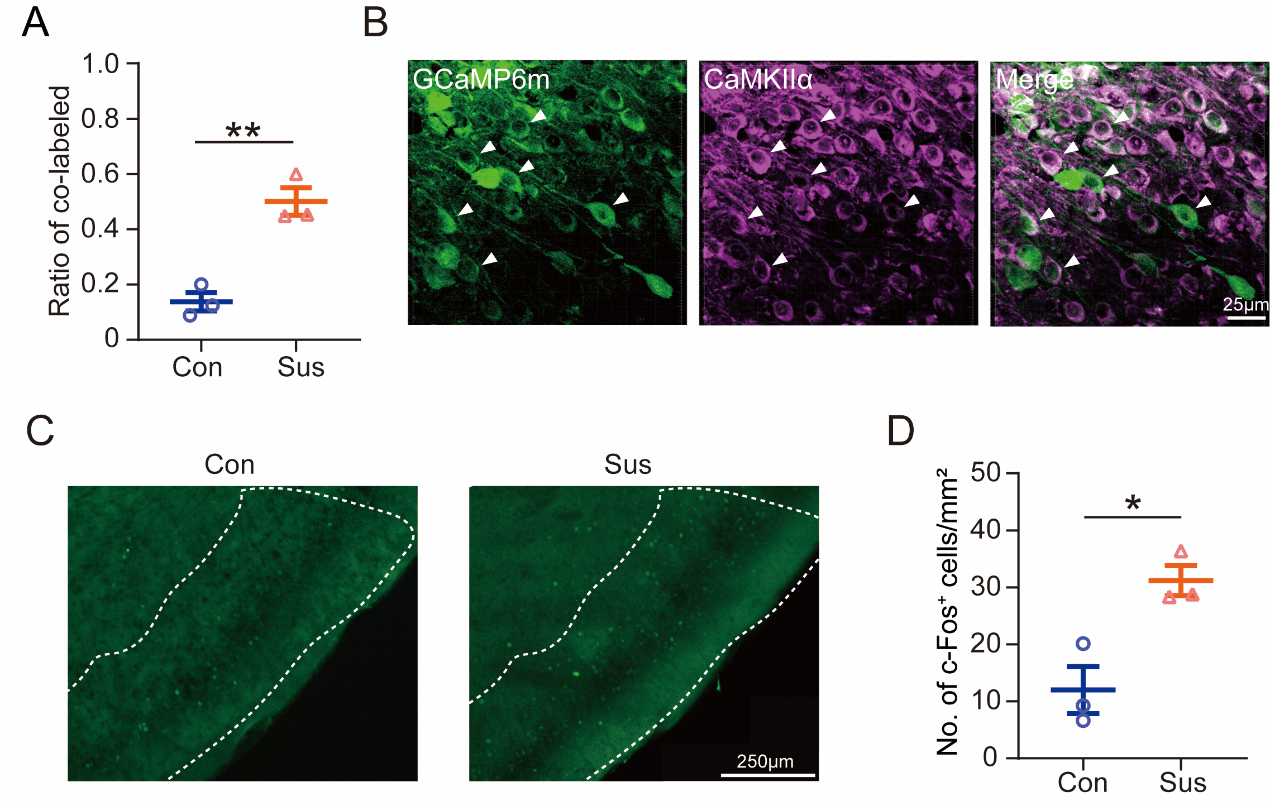


**Supplementary Figure 4. GCaMP6m virus identification and immunofluorescence images of c-fos expression in PC after MWM test. (A)** The ratio of c-Fos and CaMKIIα colocalized cells in CaMKIIα single labeled cells in PC of control and susceptible mice (*t*_4_ = 6.084, *p* = 0.0037; Unpaired Student’s t-test). Con, control, n = 3; Sus, susceptible, n = 3. **(B)** Double-staining of GCaMP6m (green) and CaMKIIα (red) and the corresponding merge images in PC of AAV2/9-CaMKIIα-GCaMP6m injection mice. The white arrowheads indicate the colocalized cells in the GCaMP6m and CaMKIIα neurons. Scale bar, 25 μm. **(C)** Control (left) and susceptible (right) mice were sacrificed 90 min after MWM to detect c-Fos expression. Scale bar, 250 μm. **(D)** Mean density of the c-Fos positive neurons in the PC of control (left) and susceptible (right) (*t*_4_ = 3.923, *p* = 0.0172; Unpaired student’s t-test). Con, control, n = 3; Sus, susceptible, n = 3. Data are presented as the mean ± SEM. **p* < 0.05, ***p* < 0.01.


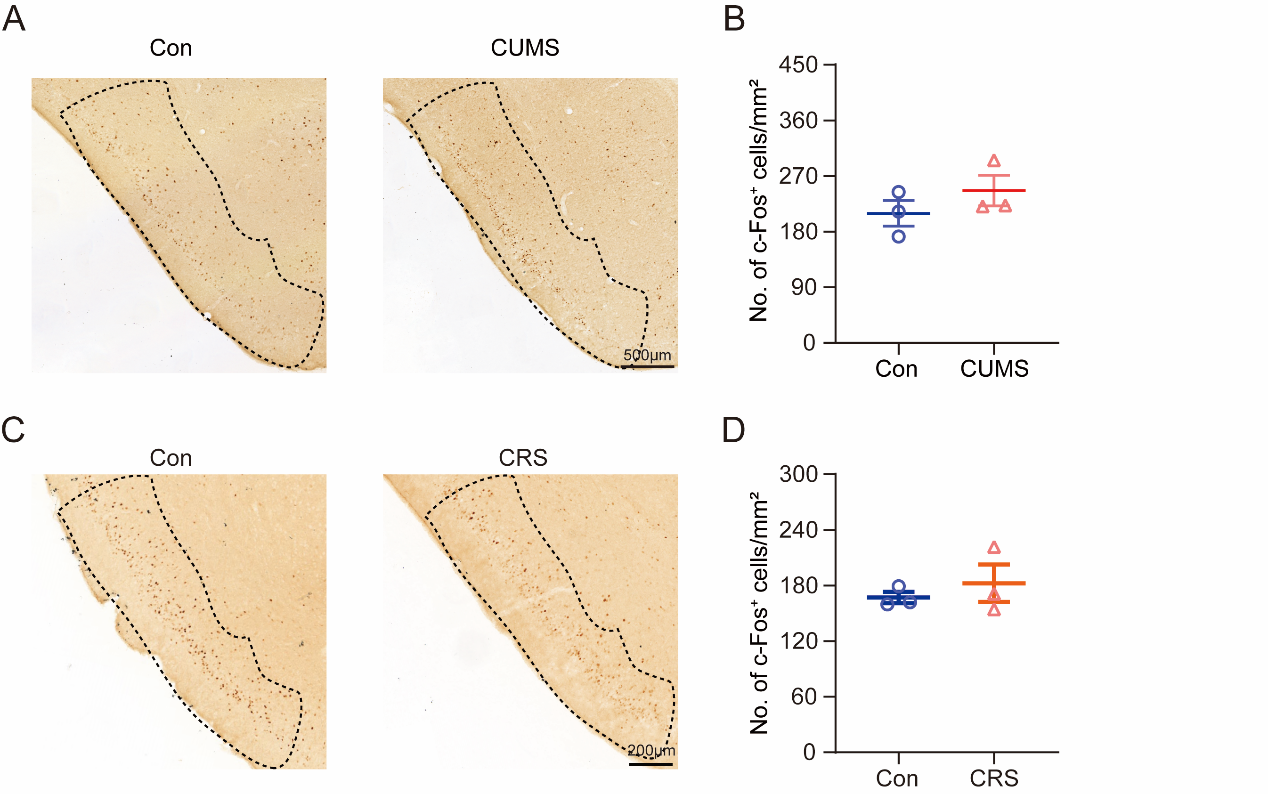


**Supplementary Figure 5. c-Fos expression of PC after MWM test exposed to CRS and CUMS.**

**(A)** Immunohistochemistry staining showing the expression of c-Fos positive cells in PC of control (left) and CUMS (right) mice. Scale bar, 200 μm. **(B)** Mean density of the c-Fos positive neurons in the PC of control (left) and CUMS (right) mice (*z* = -1.807, *p* = 0.071; Wilcoxon rank sum test). Con, control, n = 3; CUMS, chronic unpredictable mild stress, n = 3. **(C)** Representative image of cells with control (left) and CRS (right) mice. Scale bar, 200 μm. **(D)** The mean density of c-Fos positive cells in the PC (*t*_4_ = 0.7232, *p* = 0.5096; Unpaired student’s t-test). Con, control, n = 3; CRS, chronic restraint stress, n = 3. Data are presented as the mean ± SEM.


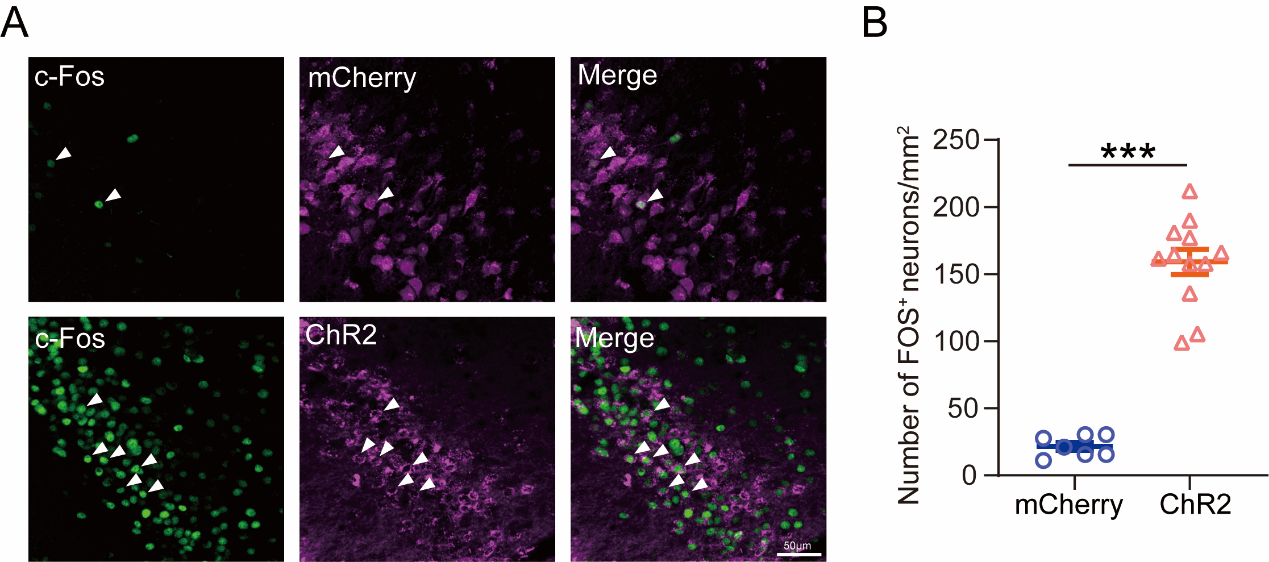


**Supplementary Figure 6. ChR2 cells were coexpressed with c-Fos.**



**(A)** Double staining of ChR2 (green) and c-Fos (red) and the corresponding merge images in the PC. The white arrowheads indicate the colocalized cells expressing both c-Fos and CaMKIIα neurons. Scale bar, 50 μm. **(B)** The mean density of c-Fos positive neurons in the PC following light stimulation (*t*_17_ = 10.87, *p* < 0.001; Unpaired Student’s t-test). mCherry, injection with mCherry-expressing viral vector in mice, n = 7, mice = 3; ChR2, injection with ChR2-expressing viral vector in mice, n = 12, mice = 3. Data are presented as the mean ± SEM. ****p* < 0.001.



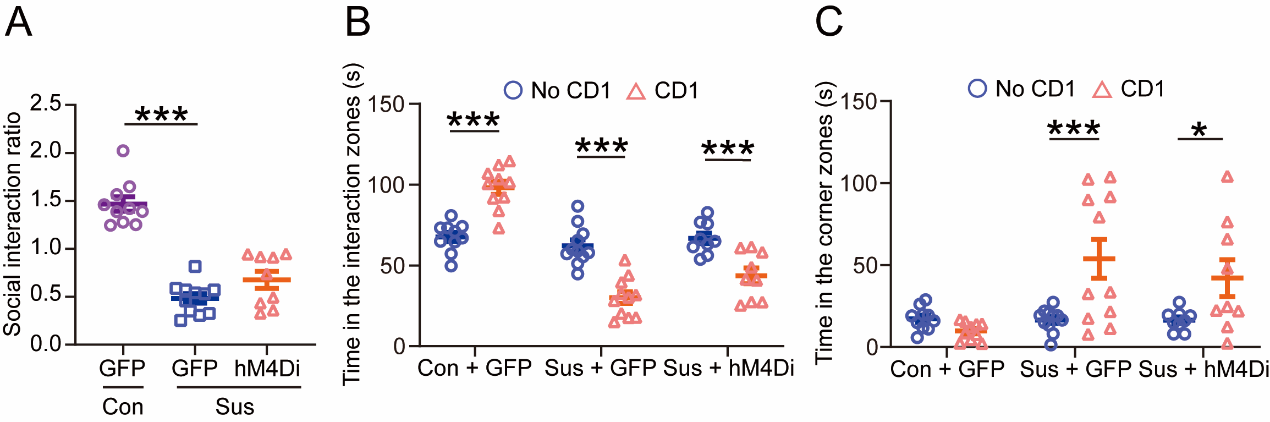


**Supplementary Figure 7. The susceptible mice appear to have social defeat.**

**(A)** The interaction ratio is displayed for control-GFP, susceptible-GFP mice, and susceptible-hM4D(Gi) mice (*F* _(2,27)_ = 55.98, Con + GFP vs Sus + GFP: *p* < 0.001; Con + GFP vs Sus + hM4Di: *p* < 0.001; One-way ANOVA). Con + GFP, injection with GFP-expressing viral vector in control mice, n=10; Sus + GFP, injection with GFP-expressing viral vector in susceptible mice, n = 11; Sus+hM4Di, injection with hM4D(Gi)-expressing viral vector in susceptible mice, n = 9. **(B)** Time in the interaction zone in the absence or presence of the CD1 (Interaction: *F* _(2,54)_ = 41.23, Con + GFP: *p* < 0.001; Sus + GFP: *p* < 0.001; Sus+hM4Di: *p* < 0.001; Two-way ANOVA). Con + GFP, n = 10; Sus + GFP, n = 11; Sus+hM4Di, n = 9. **(C)** The time spent in the corner zone of absence or presence of the CD1 (Interaction: *F* _(2,54)_ = 5.742, Con + GFP: *p* > 0.999; Sus + GFP: *p* < 0.001; Sus + hM4Di: *p* = 0.0477; Two-way ANOVA). Con + GFP, n = 10; Sus + GFP, n = 11; Sus+hM4Di, n = 9. Data are presented as the mean ± SEM. **p* < 0.05; ****p* < 0.001.
